# The Methodology of Doppler-Derived Central Blood Flow Measurements in Newborn Infants

**DOI:** 10.1155/2012/680162

**Published:** 2012-01-16

**Authors:** Koert A. de Waal

**Affiliations:** ^1^University of Newcastle, Newcastle, NSW 2305, Australia; ^2^Department of Neonatology, John Hunter Children's Hospital, Lookout Road, New Lambton, NSW 2305, Australia

## Abstract

Central blood flow (CBF) measurements are measurements in and around the heart. It incorporates cardiac output, but also measurements of cardiac input and assessment of intra- and extracardiac shunts. CBF can be measured in the central circulation as right or left ventricular output (RVO or LVO) and/or as cardiac input measured at the superior vena cava (SVC flow). Assessment of shunts incorporates evaluation of the ductus arteriosus and the foramen ovale. This paper describes the methodology of CBF measurements in newborn infants. It provides a brief overview of the evolution of Doppler ultrasound blood flow measurements, basic principles of Doppler ultrasound, and an overview of all used methodology in the literature. A general guide for interpretation and normal values with suggested cutoffs of CBFs are provided for clinical use.

## 1. Introduction

**Figure 1:**
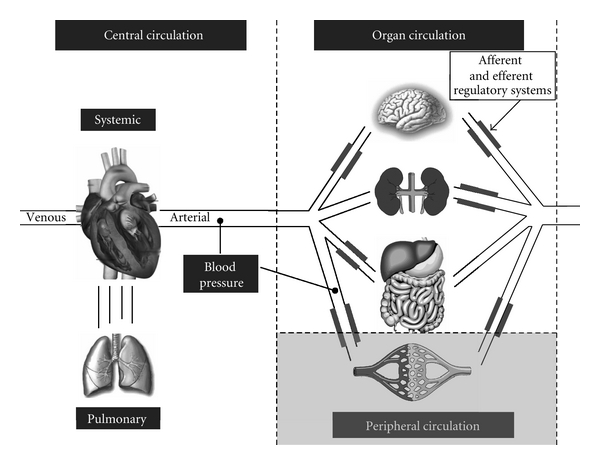
Compartments of the circulation where blood flow can be measured. The central circulation includes the pulmonary and systemic circulation. Organ circulation includes each organ and the peripheral circulation. All organs have their local afferent and/or efferent regulation system. Blood pressure in newborns is measured in the central circulation (descending aorta) or in the peripheral circulation (limbs).

Central blood flow (CBF) measurements are measurements in and around the heart. It incorporates cardiac output, but also measurements of cardiac input and assessment of intra- and extracardiac shunts. Schematically presented in Figure 1, CBF can be measured in the central circulation as right or left ventricular output (RVO or LVO) and/or as cardiac input measured at the superior vena cava (SVC flow). Intra- and extracardiac shunts like the foramen ovale, atrium or ventricular septal defects, or ductus arteriosus are measured in the central circulation to assist in interpreting RVO and LVO. Organ blood flow can be measured in the arterial blood vessels of the main organs. Peripheral blood flow, as part of the organ circulation, refers to blood flow in the skin, mucus membranes, or underlying muscle tissue. 

As CBF mainly measures cardiac output and cardiac input, some basic principles of blood flow regulation need to be addressed. Although details of the circulation are complex, there are three basic principles that underlie all functions of the system [1].

1. The blood flow to each tissue of the body is controlled in relation to the tissue needs. When tissues are active, regional flow can be increased up to 30 times the resting level. Cardiac output can only be increased several times, so blood flow must also be controlled at the local level, which will result in redistribution of total blood flow. Only the increase in total blood flow can be measured with CBF measurements, not the level of distribution in the organ circulation.
2. Cardiac output is controlled mainly by the sum of all the local tissue flows. The heart acts as an automaton; cardiac input determines cardiac output. It facilitates the use of cardiac input measurements as representative for cardiac output. The input = output response is not always sufficient, with the autonomic nervous system playing an important role in maintaining adequate output in disease states by altering heart rate and peripheral vascular resistance.
3. Arterial blood pressure is controlled independently of either local blood flow control or cardiac output control. This will affect the physiological relationship between pressure and flow. Flow (*Q*) through a blood vessel is determined by the pressure difference (Δ*P*) and the vascular resistance (*R*), *Ohms' law, *

(1) $$\begin{array}{c} Q\;=\;\frac{\Delta P}{R} \end{array}.$$

Resistance is proportional to vessel radius (*r*), vessel length (*L*), and blood viscosity (*η*), where viscosity is mainly determined by blood haemoglobin content. The expression for resistance can be combined with the equation describing the relationship between flow, pressure, and resistance in the *Poiseuille's equation:*

(2) $$\begin{array}{c} Q\;=\;{\pi \cdot r}^{4}\cdot \frac{\Delta P}{8\cdot \eta \cdot L}. \end{array}$$

In this equation, the resistance of a vessel is in proportion to the fourth power of its radius, making the vessel diameter the most contributing factor to blood flow.

## 2. Doppler Ultrasound

CBF can be measured using several techniques, for example, using oxygen consumption as a determinant of flow (using the Fick principle), dilution methods using dye or hot or cold fluid boluses, pulse pressure methods, Doppler ultrasound, or velocity-encoded phase contrast MRI. An extensive review of available methods and their respective advantages and limitations is published elsewhere [2]. The Doppler principle as applied to ultrasound describes the shift in frequency of the returning sound wave in proportion to the velocity of the object imaged. Doppler ultrasound is used to detect and measure blood flow with the major reflector being the red blood cell. Velocity of moving blood (*V*) is then calculated using the Doppler shift (*Df*), insonating frequency (*f*), speed of sound (*c*), and the angle of insonation (cos⁡⁡*q*, the angle between the sound waves and direction of moving blood) in the *Doppler equation:*

(3) $$\begin{array}{c} V\;=\;\frac{Df\cdot c}{2\cdot f\cdot \cos q}.\; \end{array}$$

Appropriate angle of insonation (also called Doppler angle) is essential for accurate determination of Doppler shift and blood flow velocity, with an increasing angle causing progressive underestimation of flow velocity.

Blood flow throughout most of the central circulation is laminar, characterised by a flow profile that is parabolic. This occurs in long, straight blood vessels under steady flow conditions. The practical implication of parabolic laminar flow is that when flow velocity is measured using pulsed Doppler, the velocity represents the average velocity of the cross-section of the vessel. Plotting the average velocity against time allows for calculation of the velocity time integral (Vti), the area under the velocity envelope.

Blood flow (*Q*) is calculated using the following parameters: Diameter (*d*) to calculate the cross-sectional vessel area (*π* · *d* ^2^/4) assuming that the vessel area is round, flow velocity (Vti), and heart rate. The following formula is used to calculate flow in mL/kg/min:

(4) $$\begin{array}{c} Q\;=\;\frac{\text{Vti}\cdot \;\text{Heart}\;\text{rate}\cdot \left({\pi \cdot d}^{2}/4\right)}{\text{body}\;\text{weight}} \end{array}.$$

The diameter can be measured using two-dimensional (2D) images from one or two ultrasound imaging planes, or one can use the M mode technique. M mode records motion of tissue toward and away from the transducer and has the advantage of producing a clear delineation of vessel walls. The disadvantage of M mode is the potential of tangential cuts through the vessel, producing an overestimation of vessel diameter.

Doppler can also be used to estimate pressure. The pressure difference (Δ*P*) over a cross-sectional area can be calculated by using the maximum velocity (*V*) in the *modified Bernoulli equation:*

(5) $$\begin{array}{c} \Delta P\;=\;{4\cdot V}^{2} \end{array}.$$

With laminar flow, there is a linear relationship between perfusion pressure and flow. This relationship weakens if flow becomes turbulent, with more perfusion pressure needed for a given flow.

In general, Doppler measures of blood flow in the central circulation have been shown to have good inter- and intraobserver reproducibility and to be well correlated with invasive measures of blood flow using microspheres, dye dilution, or thermodilution techniques. Doppler was regarded as most useful when used to track changes [3]. 

Potential errors of measurements come from inaccurate measurement of vessel diameter due to poor views or tangentially performed measurements, or inaccurate measures of Vti due to a high angle of insonation or placement of the Doppler gate out of the laminar stream.

Measuring CBF in newborns using Doppler ultrasound is performed in 2 steps. First, image acquisition (usually at the bedside) followed by image analysis using the incorporated software of the ultrasound equipment. Image acquisition in newborn infants requires a high-resolution ultrasound (US) machine and high-frequency Doppler probe with colour mode incorporated. Image analysis is the manual tracing (Vti's) and determination of distances using callipers (vessel diameter and heart rate) for the calculation of CBF.

Since the introduction of US in cardiac medicine in the late seventies, commercial US machines are now in its fourth generation. First-generation US equipment included only M mode measurements of ventricular dimensions and unguided Doppler measurements using a pencil probe. A survey by Sahn in 1978 suggested that major problems existed with M mode interobserver variability [4]. It was difficult to compare echocardiographic data from one laboratory compared with the results from another due to differences in timing of M mode measurements (e.g., at onset or at peak of QRS) and in actual distances taken (e.g., leading to leading edge or trailing to leading edge dimensions). The paper presented new recommendations for uniform M mode measurements. When 2D imaging became widely available in the late seventies (second-generation US equipment), this led to an increase in publications describing the use of cardiac ultrasound and central blood flow measurements. A report of the American Society of Echocardiography Committee on Nomenclature and Standards in 2D Echocardiography brought uniformity in transducer location, imaging planes, and image orientation standards [5]. 

Further refinement of US equipment included higher spatial resolution with increased Doppler frequency and a greater variety in size and shape of US probes (third-generation US equipment) and development of software for advanced measurements like tissue Doppler, strain rate, 3D visualisation with miniaturisation of US probes, and US equipment (fourth-generation US equipment). It is clear that early pioneer work is invaluable; however, we must realise that the normal values produced with first- and second-generation US equipment are difficult to compare to values produced with current-generation US equipment. 

## 3. Methodology of Doppler Derived Central Blood Flow Measurements

In newborn infants, CBF is measured at 3 sites: right ventricular output (RVO) at the pulmonary valve, left ventricular output (LVO) at the aortic valve, and flow returning to the heart in the superior vena cava (SVC) measured at the point where the SVC starts to open up into the right atrium [6–8]. As mentioned before, additional sites of measurements help in correct interpretation of the CBF values. Systemic blood flow is defined as the proportion of central blood flow directed to the organs of the body, and pulmonary blood flow as the proportion of CBF directed to the lungs. RVO has traditionally been associated with pulmonary blood flow and LVO with systemic blood flow. However, the presumed associations of either parameter are not true if large shunts are present. Referring back the basic principles of cardiac output regulation, LVO reflects all pulmonary venous blood returning to the left side of the heart (systemic blood flow and ductal left-to-right shunt) and RVO is the resultant of cardiac input and the often left-to-right shunt over the foramen ovale. At a time when shunting is prevalent, usually in the first few days of life (early transition), RVO would better reflect systemic blood flow and LVO would better reflect pulmonary blood [9]. Once significant shunting no longer takes place, LVO would represent systemic blood flow and RVO pulmonary blood flow. SVC flow, as a measure of cardiac input, could partly reflect systemic blood flow, as it reflects blood returning from the upper body and brain, but not blood returning from the lower part of the body. Kindly check the enumeration of all sections and subsections. Please check.

This paper will discuss the methodology of Doppler-derived LVO, RVO, SVC flow, ductal assessment, foramen ovale assessment, and flow in the descending aorta (DAo). Unfortunately, blood flow in the inferior vena cava (IVC) is difficult to measure via a transthoracic approach due to the small common confluence and its anatomical position for flow velocity determinations. The same can be said for pulmonary venous blood flow. 

### 3.1. Left Ventricular Output (LVO)

**Figure 2:**
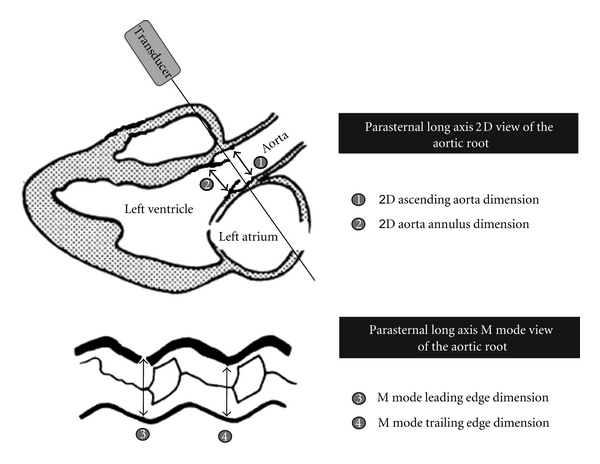
Methods of determination of LVO diameter. 2D ascending aorta dimensions are inner wall dimensions. 2D aortic annulus dimensions are taken at the valve hinges. Trailing edge technique measures the inner diameter of a vessel in M mode from the posterior portion of the anterior aortic wall to the inner boundary of the posterior aortic wall. Leading edge technique measures from the anterior portion of the anterior aortic wall to the inner boundary of the posterior aortic wall.

LVO diameter is obtained in the parasternal long axis view, and flow velocity from the subcostal to apical view or the high suprasternal view. LVO measurements using Doppler ultrasound in newborns were first published by Alverson et al. [10]. They used M mode to determine the diameter of the ascending aorta and a high suprasternal continuous wave Doppler position to obtain LVO flow velocity in 8 preterm and 14 term newborns in the first week of life. They reported a mean (SD) LVO of 221 (56) and 236 (47) mL/kg/min, respectively. Walther et al. investigated a larger group of term and preterm infants, using slightly different methodology [6]. The leading edge technique was used instead of the trailing edge technique (see Figure 2 for details). Comparable LVO values were reported for term infants, and higher mean (SD) values of 260 (35) mL/kg/min were found for preterm infants. For clinical use, 325 and 200 mL/kg/min were reported to be used as upper and lower limits of normal. Mandelbaum-Isken introduced the use of the apical window for flow velocity determinations in newborn infants [11]. A much lower LVO of 150 (40) mL/kg/min was found using the apical window and the (smaller) aorta annulus in 18 healthy term newborns. Most current research on LVO in preterm infants uses the methodology and proposed lower limit of 150 mL/kg/min for clinical use as described by Evans and Kluckow [7]. They used the internal diameter from 2D images of the ascending aorta and the apical window for flow velocity to calculate LVO. Mellander et al. compared the different methods to measure the LVO diameter, including 2D measurements at the aortic annulus [12]. They found that aortic root diameters often overestimated and 2D aortic annulus underestimated LVO as compared to thermodilution. The aortic root is an area stretching from the aortic annulus to the proximal ascending aorta, including the sinuses of Valsalva and the supra-aortic ridge. M mode measurements do not prespecify what area is actually measured. If the recommendations by Sahn et al. [4] are followed, then is it likely that the widest area at the sinuses of Valsalva is measured. There are considerable differences in diameter of the aortic annulus and sinuses of Valsalva in children and adults but limited data is available for the newborn population [13, 14]. In preterm infants, the aorta annulus is approximately 0.9 mm smaller than the ascending aorta, decreasing the LVO by 100 mL/kg/min [15]. 

The methodology of Doppler determination of LVO and the reported values in newborn infants is presented in Table 1. Variation exists in diameter location (ascending aorta versus aortic root or aorta annulus), in methodology of diameter determination (M mode versus 2D, leading edge versus trailing edge technique), in Doppler method (continuous Doppler, CW versus pulse wave Doppler, PW), and in location of flow velocity determinations (suprasternal versus subcostal or apical). Most studies did not use angle correction for flow velocity. However, the anatomic position of the left ventricular outflow tract in newborn infants is seldom truly aligned (in 3D geometry) with any apical or subcostal view, creating an underestimation of the true LVO.

The accuracy of LVO measurements as compared to the Fick method, thermodilution or dye dilution varies between 1 and 36% [3]. Hudson et al. evaluated intra- and interobserver agreement in 20 healthy term infants, using 3 different methods for aortic diameter measurements and 3 different sites of measuring blood flow velocity with both continuous wave and pulsed Doppler [26]. In this study, the most reproducible determination of cardiac output was found when the suprasternal site with continuous wave Doppler was used for measurement of blood flow velocity and M mode trailing edge technique was used for diameter.

More recently, Tsai-Goodman et al. reported on repeatability of measures of LVO in term and preterm infants with no significant differences within or between observers for any of the parameters required to measure LVO [22]. It is possible that improved image quality of current US equipment also improved intra- and interobserver agreement. 

### 3.2. Right Ventricular Output (RVO)

RVO diameter and flow velocity are obtained by a true parasternal view, looking slightly upwards to align with the right ventricular outflow tract. Doppler determinations of RVO in newborns were first published in 1987 by Takenaka and Sholler, but did not receive much clinical attention until later reports by Evans et al. [7, 27, 28]. The right ventricular outflow tract and pulmonary valve lie very close to the anterior chest wall, making Doppler measurements easy. Most studies use the parasternal view to visualise the pulmonary annulus for diameter determinations in end systole, using the hinges of the pulmonary valve as reference point. Flow velocity can be measured using the same view. Some investigators use the short-axis view to obtain the same parameters (Table 2). Diastolic flow and/or turbulent flow from ductal shunting can make flow velocity measurements difficult, as precise tracing of the pulmonary waves is not always possible. The accuracy of Doppler RVO is not known, as there are no publications comparing Doppler RVO versus other methods of right-sided cardiac output determinations. The intra- and interobserver agreement has been determined by Tsai-Goodman et al. with the major difference found being measurements of the pulmonary outflow tract diameter [22]. Intraobserver repeatability was 4%, 7.5%, and 9%, respectively, for measurements of the hinges of the pulmonary valve, pulmonary trunk, and right ventricular outflow tract. There were significant differences between observers for measurement of the pulmonary trunk and right ventricular outflow tract, but not for the hinges of the pulmonary valve. The mean RVO was 255 mL/kg/min with a mean difference between observers of only 0.3 mL/kg/min (95% CI: −24.1 to 23.4 mL/kg/min).

### 3.3. Superior Vena Cava Flow (SVC Flow)

SVC flow is a relative new method of measuring central blood flow. It measures blood flowing back to the heart from the upper body and brain and is not influenced by atrial or ductal shunting. Its use in newborns was first described by Tamura et al. [31]. They sequentially measured 17 healthy term infants in the first day of life, exploring the maximum venous flow velocity during ventricular systole (S wave) and diastole (D wave). Five to 7 cycles in expiration were used to average the flow velocity. They did not measure SVC diameters. The most used methodology for measuring SVC flow is the methodology presented by Kluckow and Evans [8]. They measured SVC flow in 25 preterm and 14 term infants, using the high parasternal view rotated towards the true sagittal plane for diameter measurements. SVC image acquisition can be difficult, especially in spontaneous breathing infants. It is especially important to obtain the full diameter, as the SVC can “hide” behind the ascending aorta. The minimum and maximum diameters were taken at the point where the SVC starts to open up into the right atrium and averaged from 3 to 5 cardiac cycles. Flow velocity was measured from the low subcostal view with the probe directed towards the SVC. Since SVC flow is venous flow, the beat to beat variability is of importance. Spontaneous respiration will influence flow velocity, therefore it is advised to take at least 10 to 15 cycles to average flow velocity Table 3.

Kluckow and Evans reported that in infants with a closed duct, where LVO and RVO equate to systemic blood flow (assuming there is no significant FO shunt), SVC flow was an average 37% of LVO [8]. The median intraobserver variability for SVC flow measurement was 8.1%, and the median interobserver variability between the measurements was 14%. Measurement of velocity time integral (median variability 7.4%) and diameter (median variability 8.7%) contributed more to the variability than heart rate (median variability 1.8%). Groves et al. showed comparable findings on intra- and interobserver variability [32]. Lee et al. investigated the image quality and intra- and interobserver agreement of SVC flow. Reliable diameter images were obtained in 85% and velocity recordings in 81% of the patients, reflecting difficulties in image acquisition [33]. The mean variability of SVC flow in this study was 17% in the intraobserver analysis and 29% in the interobserver analysis.

### 3.4. Assessment of the Ductus Arteriosus (DA)

The ductus arteriosus is a pulmonary to systemic shunt in fetal life, where it carries most of the RVO. Shortly after birth, the shunt reverses due to an increase in systemic vascular resistance (release from the low-resistance placental circulation) and a decrease in the pulmonary vasculature resistance (lung inflation). The shunt becomes systemic to pulmonary (left to right, LR) as long as systemic pressure is higher than the pulmonary pressure throughout the cardiac cycle. Normally the DA closes soon after birth and the shunt disappears, but this process is often delayed in very preterm and sick newborns.

Ductal shunting will influence central blood flow, mainly LVO, as most of the volume of shunt will be directed left to right. With significant right-to-left (RL) shunt, often due to high pulmonary vascular resistance and hence decreased pulmonary blood flow, ductal shunting can be associated with reduced LVO but with a normal venous return from the lower body.

It is important to include ductal assessments in central blood flow measurements to be able to interpret the findings. Ductal assessment should include at least ductal diameter, maximum LR flow velocity (*V*_max⁡_) and flow pattern (continuous, pulsatile, bidirectional including % RL shunt, that is, the amount of time of the cardiac cycle blood flows right to left). Several other ultrasound measurements are suggested to help determine the degree of shunting. They include the ratio between the dimensions of the left atrium and the aorta (LA/Ao ratio) [34], left pulmonary artery diastolic velocity (LPAd) [35], and measuring the flow pattern of the descending Aorta (DAo) [24], the cerebral arteries [36, 37], or the abdominal organ arteries [38–40]. 

Ductal diameter is probably the most important parameter to determine the degree of ductal shunting. As with any flow, the diameter will have the largest impact on the amount of flow. Commonly, the duct is wide on the aortic side with constriction starting at the pulmonary site of the duct. To capture this aspect, one should visualise the whole trajectory of the duct. Most investigators measure the DA diameter from the high left parasternal view, with optimised colour flow Doppler mapping scale and gain settings. Care should be given to prevent colour spill from excess gain. The minimum diameter (site of maximal constriction) of the colour flow jet closest to the entry to the main pulmonary artery is then analysed through frame by frame analysis, and the diameter is taken at the clearest appearance in end systolic frames [41, 42]. The coefficient of variation using this methodology was 12% [41]. As the colour jet of a ductus arteriosus will widen out into the pulmonary trunk, it is essential to locate the site of maximal constriction. 

Table 4 shows studies investigating ductal diameter in a wide variety of preterm infants and the ranges found. Median diameter is dependent on postnatal age with earlier measurements (within 12 hours of life) usually showing larger diameters.

With current generation ultrasound equipment, it has become increasingly easy to measure the internal diameter of the duct using 2D images. The short axis view to measure ductal diameter is not preferred as it will not always visualise the whole trajectory of the duct and commonly visualise the (wider) ductal jet without showing the site of maximal constriction. Constricting ducts can change shape and show as tortuous or kinked, making it difficult to find the site of maximal constriction.

For interpretation of central blood flow measurements, a ductal diameter greater than 1.5-1.6 mm can decrease SVC flow during transition, increase LVO, and decrease flow in the descending aorta [24, 42, 44, 46]. Evans and Kluckow evaluated the effect of various cardiorespiratory factors on RVO and LVO in 120 ventilated preterm infants [7]. A significant ductal shunt resulted in an increased LVO to RVO ratio. Up to 37% of ventilated preterm infants had suboptimal systemic blood flow which would not be detected if only LVO was measured.

Doppler evaluation at the point of DA diameter measurement will provide the ductal flow pattern. It is important to measure velocity and pattern in the duct itself at its narrowest point and not near the aortic side of the duct or in the ductal jet in the main pulmonary artery. A ductal flow pattern can be left to right (LR), bidirectional (BD), or right to left (RL) depending on the pressure difference between the two ends. Pure RL shunt is always pathological in newborn infants, but a small degree of BD shunting is normal shortly after birth [56]. When the pattern is bidirectional, the proportion of the cardiac cycle with right-to-left shunting could be measured as the time of right-to-left shunting divided by the total length of the cardiac cycle. Assuming that the systemic pressure is normal, a RL ductal shunt percentage of more than 30% is often considered significant pulmonary hypertension. 

Su et al. classified ductal Doppler flow patterns on visual appearance into 5 categories; pulmonary hypertension pattern, growing pattern, pulsatile pattern, closing pattern, and closed pattern [57]. Interobserver agreement to classify the patterns was not tested. The pulsatile pattern showed the highest specificity (100%) and sensitivity (93.5%) to predict a persisting DA with clinical signs. The closing pattern, commonly with a flow velocity > 200 cm/s and a continuous appearance, was associated with constriction.

True ductal flow incorporating ductal diameter and Vti's has been performed in a few studies [58, 59]. Although ductal flow is initially laminar, constriction will often change the flow to turbulent making representative tracings of flow velocity difficult.

### 3.5. Assessment of Shunt over the Foramen Ovale (FO)

The FO is an area in the midportion of the atrial septum concerning the fossa ovalis. In fetal life is has a function as divider for blood into the right or left side of the heart. The edge of the atrial septum (crista dividends) divides the incoming flow in two arms. Flow from the ductus venosus is diverted predominantly in the left atrium, and blood flow from the inferior vena cava enters the right atrium [60]. After birth, when atrial and ventricular pressures change, the valvular structure has the potential to close the defect. In the first few days of life, a shunt over the FO is common. It shows a dominant left-to-right direction and a bidirectional flow pattern [53]. 

The atrial septum can be imaged from a subcostal four-chamber view, adding colour flow Doppler mapping with colour scale setting for low velocities to assess shunts across the septum. The diameter can be measured using the color flow jet across the septum [54] or by using 2D images [54, 55]. The pulsed wave Doppler gate is placed in the interatrial shunt at the level of the atrial septum to determine flow direction and flow velocity. The pattern of flow should then be classified as left to right, bidirectional, or right to left. When the pattern is bidirectional, the proportion of the cardiac cycle with right-to-left shunting could be measured as the time of right-to-left shunting divided by the total length of the cardiac cycle as described for ductal flow patterns.

An FO shunt can influence central blood flow measurements, with its main effect increasing RVO. Evans and Iyer reported studies in 51 ventilated preterm infants with an increasing RVO to LVO ratio if the FO diameter exceeded 3 mm [53, 61]. Only few of these large FO shunts persisted during admission, making the FO shunt less likely to influence central blood flows compared to ductal shunting.

The natural course and closure of the FO is influenced by the presence of a DA and gestational age. In a study by Riggs et al. evaluating the natural course of an FO in 80 term and preterm infants, younger gestational age was associated with delayed closure and the presence of a DA at the time of initial diagnosis of an FO was strongly associated with earlier closure of the FO [55].  Table 5 shows studies investigating the FO in newborn infants.

### 3.6. Descending Aorta Flow (DAo Flow)

To facilitate measurements of blood flow in lower body, Groves et al. designed a method to measure blood flow in the descending aorta, just proximal to the diaphragm [32]. Flow velocity was measured from a subcostal sagittal view and from the high parasternal view with PW Doppler, with the use of angle correction. Reverse flow was deducted from forward flow to create total DAo flow. The diameter of the descending aorta was measured using M mode trailing edge technique in the parasternal short axis view. Intra- and interobserver variability were 14% and 11%, respectively, with the subcostal approach showing better repeatability. Normal values were determined in 14 healthy preterm infants and 13 term infants. Median (range) DAo flow at 24 hours of age was 180 (93–233) for term infants and 133 (108–305) mL/kg/min for preterm infants. In a larger preterm population of less than 31-week gestation infant, the median DAo flow was 145 (29–255) mL/kg/min. DAo flow reversal showed significant correlations with an increased ductal shunt [24]. 

### 3.7. LVO to RVO Ratio

The LVO to RVO ratio can describe if significant shunting takes place between systemic and pulmonary system. The ratio does not discriminate where the shunt takes place (atrial or DA) and measurement errors can be multiplied when using ratios. Normal Doppler-derived LVO to RVO ratio in preterm infants is not always one, even if shunts are not present. The anatomical positions of both outflow tracts are not always measurable with less than 20° angle leading to a variable amount of underestimation of the true cardiac output. The angle is usually greater for LVO then it is for RVO.

## 4. Central Blood Flow Measurements: Clinical Use and Interpretation

Central blood flow measurements reflect global cardiac function (preload, contractility, and afterload) by measuring total blood flow through the pulmonary and/or systemic circulation. Blood flow reflects the transport of oxygen to the tissues and is determined by the demand. Often, all central blood flow parameters are high or all parameters are low. It is preferable to measure central blood flow at more than one site as a cross-check to exclude measurement errors. If a single parameter is low or high, then further exploration should take place to find the cause. This should include a full sequential segmental chamber analysis to rule out structural abnormalities and evaluation of shunts and ventricular failure with severe dilatation and subsequent obstruction of an outflow tract as found in severe pulmonary hypertension or prolonged mechanical ventilation using high distending pressures. Central blood flow measurements are probably most informative if followed over time.

Research in neonatal hemodynamics has extensively studied the transitional circulation using CBF measurements. For a review of the current concepts of transitional circulation, I refer to excellent articles elsewhere [62–65]. In summary, blood flow decreases and blood pressure rises in the first day after birth in healthy term and late-preterm infants. In contrast, very preterm infants show a rise in blood flow (and blood pressure) in the first week of life. In sick and immature infants, blood pressure and/or blood flow often show a decrease in the first hours after birth with its nadir at 5 to 12 hours after birth. It has been shown that this period of low systemic blood flow at the most vulnerable period is associated with mortality and poor neurodevelopmental outcome [66, 67]. Risk factors for developing low systemic blood flow are a very young gestation, steal from blood out of the systemic circulation via a large ductus arteriosus, the use of mechanical ventilation, and severe respiratory disease. Most studies use an SVC flow < 45 mL/kg/min or LVO or RVO < 150 mL/kg/min as the definition of low flow, with SVC flow the best studied parameter in relation to outcome.  Table 6 presents mean and standard deviation of CBF values in term and preterm infants using the methodology as described with adjusted diameters for LVO for preterm infants at day 7 to 14 [7, 8, 15, 25, 68].  Table 7 provides suggested cutoffs for low and high central blood flow in preterm infants based on the available evidence.

## 5. Future Perspectives and Directions for Research

Central blood flow measurements are increasingly used in clinical neonatology [69]. Its importance in clinical decision making in specific situations has been described [70, 71], but some argue that its importance in clinical decision making is not sufficiently studied. However, many currently used imaging techniques and diagnostic tests in neonatal medicine (including blood pressure monitoring) have not been subjected to randomized trials to determine its efficacy on outcome [69]. CBF measurements, as all diagnostic methods, are used to increase insight in the physiology and pathology.

Hemodynamics is an important part of neonatal intensive care. Commonly used parameters to guide treatment (e.g., blood pressure, capillary refill, lactate) are poorly associated with blood flow, and trials have shown that circulatory support treatment does not always have the expected effect on central blood flow [65]. These findings indicate the need to continue to explore neonatal hemodynamics by measuring central blood flow in the NICU [70]. 

Future studies are needed to evaluate the methodological issues still surrounding CBF measurements. The accuracy of all CBF measurements is currently being evaluated by phase-contrast MRI [72]. For clinical purposes, the interrater variability in longitudinal measurements needs to be minimised. Most variability of Doppler CBF is caused by diameter measurements. Variability could be decreased if population based diameter percentiles are used in the formula to calculate flow, instead of actual diameter measurements. This approach will also decrease accuracy, but longitudinal changes have proven to be the best predictors of morbidity and mortality, not absolute values [45].

More research is needed to study the effect of treatment on blood flow. To date, only very few randomized trials have explored the effect of cardiovascular treatment (volume, inotropes, and inodilators) on blood flow [49, 73, 74]. Randomizing at-risk babies to 2 different treatment regimes and preventing cross-over or contamination might reveal further insight into how to treat cardiovascular compromise. One could also suggest randomization at different thresholds or a combination of pressure and flow thresholds. All trials should use standardised methodology of measuring CBF to be able to compare hemodynamic outcomes.

The key to any successful trial is measuring the right outcome (short and long term) and obtaining the right diagnosis of the cardiovascular problem at hand. For example, the definition of a hemodynamic significant ductus arteriosus varies enormously amongst randomized trials investigating treatment of the duct [75]. There is a wealth of information that can be obtained with functional echocardiography and central blood flow measurements. To achieve its potential, there is a need to move it to the point of care where it is performed by the attending neonatologist and not by consulting specialists or researchers. As with most diagnostic methods, findings to date have not demonstrated that functional echocardiography affects outcomes. However, its value as a tool for assessing the rapidly changing hemodynamic status is essential to ensure future research can be translated into clinical practice.

## Figures and Tables

**Table 1:** Methods of determination of LVO diameter, flow velocity, and reported mean (SD) values in mL/kg/min.

	LVO diameter	LVO flow velocity	Angle correction	Time after birth	LVO (mL/kg/min)					
					Preterm infants			Term infants		
					*n*	Mean	SD	*n*	Mean	SD
Alverson et al., 1983 [10]	Ascending Aorta M mode trailing edge parasternal long axis	Ascending Aorta unguided CW doppler high suprasternal view	no	1–5 days	8	221	56	14	236	47

Walther et al., 1985 [6]	Aortic Root M mode leading edge parasternal long axis	Ascending Aorta unguided CW doppler high suprasternal view	no	1–5 days	59	260	35	62	230	30

Hirsimäki et al., 1988 [16]	Aortic Root M mode parasternal short axis	Ascending Aorta unguided CW doppler high suprasternal view	no	24 hours				22	273	59

Winberg et al., 1989 [17]	Ascending Aorta M mode trailing edge parasternal long axis	Ascending Aorta unguided CW doppler high suprasternal view	no	24 hours				16	187	35

Walther et al., 1990 [18]	Ascending aorta 2D internal diameter parasternal long axis	Ascending Aorta unguided CW doppler high suprasternal view	no	unknown	26	250	41	16	250	41

Agata et al., 1991 [19]	Ascending Aorta M mode leading edge parasternal long axis	Ascending Aorta 2D guided PW doppler apical view	no	24 hours				34	245	56

Mandelbaum et al., 1991 [20]	Aortic Annulus 2D internal diameter parasternal long axis	Ascending Aorta 2D guided PW doppler apical view	no	5-48 hours				18	150	40

Evans and Kluckow, 1996 [7]	Ascending Aorta 2D internal diameter parasternal long axis	Ascending Aorta 2D guided PW doppler apical view	<20°	24 hours	20	233^#^	55			

Evans et al., 1996 [7]	Ascending Aorta 2D internal diameter parasternal long axis	Ascending Aorta 2D guided PW doppler apical view	<20°	4 days	20	282^#^	60			

Pladys et al., 1999 [21]	Ascending Aorta M mode trailing edge parasternal long axis	Ascending Aorta 2D guided PW doppler subcostal view	no	24 hours	17	245*	60			

Tsai-Goodman et al., 2001 [22]	Aortic Root M mode trailing edge parasternal long axis	Ascending Aorta unguided CW doppler high suprasternal view	no	24 hours	10	241		16	241	

Murase et al., 2002 [23]	Ascending Aorta 2D internal diameter parasternal long axis	Ascending Aorta 2D guided PW doppler high suprasternal view	no	24 hours	11	144^⋀^	37			

Groves et al., 2008 [24]	Ascending Aorta 2D internal diameter parasternal long axis	Ascending Aorta 2D guided PW doppler apical view	<20°	24 hours	43	288^×^	80			

Sloot et al., 2010 [25]	Aortic Annulus 2D internal diameter parasternal long axis	Ascending Aorta 2D guided PW doppler subcostal view	no	7 days	57	296	74			

^#^Preterm infants with mild respiratory distress, *Preterm infants with normal blood pressure, ^*⋀*^Preterm nonventilated infants, and ^×^Preterm infants with ductal size < median ductal size for cohort.

**Table 2:** Methods of determination of RVO diameter, flow velocity, and reported mean (SD) values in mL/kg/min.

	RVO diameter	RVO flow velocity	Angle correction	Time after birth	RVO (mL/kg/min)					
					Preterm infants			Term infants		
					*n*	Mean	SD	*n*	Mean	SD
Takenaka et al., 1987 [27]	not done	RVOT 2D guided PW doppler parasternal short axis	no	24 hours						

Sholler et al., 1987 [28]	Pulmonary annulus at end systole in 2D parasternal short axis	RVOT 2D guided PW doppler parasternal short axis	no	14 days				25	310	70

Shiraishi et al., 1988 [29]	Pulmonary artery M mode leading edge parasternal long axis	RVOT 2D guided PW doppler parasternal short axis	no	1-2 days				10	200	

Walther et al., 1990 [18]	mean systolic diameter of the Pulmonary artery	RVOT 2D guided PW doppler parasternal short axis	no	unknown	26	254	48	16	254	48

Evans et al., 1996 [7]	Pulmonary annulus at end systole in 2D sagittal view	RVOT 2D guided PW doppler sagittal view	no	24 hours	19	202^#^	71			

Evans and Kluckow, 1996 [7]	Pulmonary annulus at end systole in 2D sagittal view	RVOT 2D guided PW doppler sagittal view	no	4 days	20	287^#^	60			

Yanowitz et al., 1999 [30]	Pulmonary annulus at end systole in 2D parasternal short axis	RVOT 2D guided PW doppler parasternal short axis	no	24 hours	20	355	40			

Yanowitz et al., 1999 [30]	Pulmonary annulus at end systole in 2D parasternal short axis	RVOT 2D guided PW doppler parasternal short axis	no	7 days	20	450	50			

Tsai-Goodman et al., 2001 [22]	Pulmonary annulus at end systole in 2D parasternal short axis	RVOT 2D guided PW doppler parasternal short axis	no	24 hours	10	255		16	255	

Groves et al., 2008 [24]	Pulmonary annulus at end systole in 2D parasternal short axis	RVOT 2D guided PW doppler parasternal short axis	no	24 hours	80	400	90			

Sloot et al., 2010 [25]	Pulmonary annulus at end systole in 2D sagittal view	RVOT 2D guided PW doppler sagittal view	no	7 days	57	429	116			

^#^Preterm infants with mild respiratory distress.

**Table 3:** Methods of determination of SVC diameter, flow velocity, and reported mean (SD) values in mL/kg/min.

	SVC diameter	SVC flow velocity	Angle correction	Time after birth	SVC flow (mL/kg/min)					
					Preterm infants			Term infants		
					*n*	Mean	SD	*n*	Mean	SD
Tamura et al., 1998 [31]	Not done	1 cm proximal of RA 2D guided PW doppler suprasternal view	no	24 hours				17		

Kluckow and Evans, 2000 [8]	2D internal minimum and maximum diameter high parasternal view	RA-SVC junction 2D guided PW doppler subcostal view	no	24 hours	25	82	40	13	76	38

Groves et al., 2008 [32]	M mode internal minimum and maximum diameter high parasternal view	RA-SVC junction 2D guided PW doppler subcostal view	no	24 hours	14	112	36	13	89	32

Lee et al., 2010 [33]	2D internal minimum and maximum diameter high parasternal view	RA-SVC junction 2D guided PW doppler subcostal view	no	24 hours				48	99	47

Sloot et al., 2010 [25]	2D internal minimum and maximum diameter high parasternal view	RA-SVC junction 2D guided PW doppler subcostal view	no	7 days	57	89	33			

**Table 4:** Range or mean (SD) of ductal diameters in preterm infants using a high left parasternal view with colour flow mapping at the site of maximum constriction.

	Inclusion criteria	*n*	Ductal diameter (mm)
Roberson and Silverman, 1994 [43]	<34 week gestation	48	2.6 (0.6)
Evans and Iyer, 1995 [41]	<1500 gram and mechanical ventilation	56	0–3.8
Kluckow and Evans, 1995 [42]	<1500 gram and mechanical ventilation	116	0–3.8
Evans and Kluckow, 1996 [44]	<1500 gram and mechanical ventilation	117	0–3.4
Kluckow and Evans, 2000 [45]	<30 week gestation	126	0–3.5
Osborn et al., 2003 [46]	<30 week gestation	128	0–4.3
El Hajjar et al., 2005 [47]	<31 week gestation	23	0–5.0/kg
El-Khuffash 2008 [48]	<1500 gram	33	0–4.1
Groves et al., 2008 [24]	<31 week gestation	80	0–3.9
Paradisis et al., 2009 [49]	<30 week gestation	90	2.0 (0.9)

**Table 5:** Studies investigating atrial shunt in newborn infants.

	Population studied	Timing	Findings
Fukazawa et al., 1988 [50]	102 term and preterm infants	Followup till closure	24% open at 1 week 13% open at 1 month median diameter 4 mm

Hannu et al., 1989 [51]	37 healthy term infants	24 hours	41% closed 57% LR shunt 2% BD shunt

Hiraishi et al., 1991 [52]	36 healthy term infants	4-5 days	53% closed 28% LR shunt 19% BD shunt

Evans and Iyer, 1994 [53]	51 preterm infants < 1500 grams with mechanical ventilation for more than 24 hours	Regular during the first 3 weeks and then on indication until discharge	46% < 3 mm, early closure 23% < 3 mm, persisting 18% > 3 mm, early closure 13% > 3 mm, persisting

Markhorst et al., 1995 [54]	20 healthy term infants	6 days	90% closed 10% LR shunt 0% RL shunt

Riggs et al., 2000 [55]	80 term and preterm infants with an atrial shunt	Followup till closure	median closure time in term infants 119 days median closure time in preterm infants 752 days initial diameter not influencing closure

**Table 6:** Mean and SD of central blood flow values using the methodology as described by Evans and Kluckow [7, 8].

	3–9 hours	24 hours	day 2	day 7–14
RVO (sagittal view)				
Preterm		260 (90)	270 (90)	430 (100)
Term		255 (60)		

LVO (ascending aorta)				
Preterm		240 (60)	260 (60)	400 (75)
Term		220 (60)		

SVC flow				
Preterm	60 (25)	80 (20)	90 (25)	90 (30)
Term	75 (25)	95 (30)	100 (30)	

**Table 7:** Suggested cutoffs for low and high central blood flow in preterm infants.

	Pathologically low blood flow	Low blood flow	High blood flow
RVO	<120	<150	>600
LVO	<120	<150	>600
SVC flow	<40	<45	>150
